# Effects of a 16-week individualized exercise intervention on physical and behavioral outcomes in two children with autism spectrum disorder: a case series

**DOI:** 10.3389/fpsyt.2026.1916839

**Published:** 2026-08-17

**Authors:** Yu Xing, Haoyan Liu, Liang Li, Xueping Wu, Qun Xiong

**Affiliations:** 1 School of Physical Education, Hainan University, Haikou, Hainan, China; 2 Hainan Provincial Key Laboratory of Sports and Health Promotion, Key Laboratory of Emergency and Trauma, Ministry of Education, The First Affiliated Hospital of Hainan Medical University, Hainan Medical University, Haikou, Hainan, China; 3 School of Physical Education and Training, Shanghai University of Sport, Shanghai, China; 4 School of Physical Education, Jiangxi Normal University, Nanchang, Jiangxi, China; 5 Department of Rehabilitation II, Affiliated Hospital of Jiangxi University of Traditional Chinese Medicine, Nanchang, Jiangxi, China

**Keywords:** autism spectrum disorder, case report, children, health-related fitness, movement development, physical activity

## Abstract

**Objective and setting:**

This study aims to explore the effects of exercise intervention on the physical and mental rehabilitation of children with ASD, and provide a reference for expanding behavioral intervention methods for autism.

**Design:**

This study used two children with autism, Case B and Case D, as research subjects. According to the physical and mental characteristics of children with ASD, a 16-week, three-stage rehabilitation program was designed and implemented. During the training period, measurement methods such as ActiGraph GT3X+, Health Related Physical Fitness (HRPF), Test of Gross Motor Development 3 (TGMD-3), and ABC Behavior Observation Scale were used to evaluate the effects of rehabilitation training at each stage.

**Results:**

After completing the targeted intervention, the physical acvtivity of the two children were decreased sedentary behavior (*M*_change_=290.07 d/min, 218.16 d/min), the increased moderate/high-intensity physical activity (*M*_change_=135.05 d/min, 36.45 d/min), In terms of healthy physical fitness level, the body mass index (BMI) of the the children with ASD was decreased (*M_change_* = 0.7, 4.92), together with the increased grip strength (*M*_change_=1.8, 4.2) and forward bending in the new sitting position (*M*_change_=4.3, 5.6). In terms of fundamental motor skills, the total score of the children with ASD was increased (*M*_change_=4, 10), especially the object control skill score (*M*_change_=4, 7). In terms of the physical behavior, the frequency and duration of stereotyped behaviors of the two children were decreased. In addition, the positive communication times of the two children were also increased (*M*_change_=14, 12).

**Conclusion:**

The 16-week exercise intervention improved the physical activity and health fitness levels of the autistic children to some extent, and meanwhile, the two children also showed some improvement in their behavior. They suggest that individualized exercise may warrant further investigation as a supportive component of educational rehabilitation, but causality and generalizability cannot be established without larger controlled studies and long-term follow-up.

## 1 Introduction

Autism is a pervasive neurodevelopmental disorder with complex etiology, which often develops in early childhood and is characterized by two core symptoms: repetitive and stereotyped behavior patterns and social communication/interaction disorders (1). According to statistics from the Centers for Disease Control and Prevention of the United States, the prevalence of autism in children has increased from 1:54 in 2020 to 1:36 in 2023 (2). In 2022, the Autism Screening and Intervention Service Specifications for Children Aged 0–6 issued by the National Health Commission showed that the prevalence of autism in children in China is about 7% (3). Consequently, the continued increase in the incidence of autism worldwide has posed a severe challenge to the country and society, which has become a major public health issue that urgently requires global attention and response.

Currently, the intervention and treatment methods used to improve the physical and mental health of autistic patients, especially children, mainly include a variety of approaches such as behavioral therapy, drug therapy, speech therapy, sensory integration training, and motor intervention. Among them, behavioral therapy (such as applied behavior analysis, ABA) has a significant effect on improving the behavioral and social skills of autistic children. However, ABA therapy requires the guidance of professionally trained behavior analysts and requires long-term, high-intensity training, which places a large time and financial burden on families. Although drug treatment is quick-acting and can relieve symptoms in a short period of time, it can have side effects (such as drowsiness, weight gain, gastrointestinal discomfort, etc.) and cannot fundamentally solve the core symptoms of autism. The intervention effects of speech therapy and sensory integration therapy vary significantly among individuals. Exercise intervention is receiving increasing attention because it is consistent with the physiological and psychological characteristics of autistic children.

To further improve the physical and mental health of autistic children from the perspective of physical education, such as improving motor abilities, improving physical activities and physical functions, and improving the interaction between individuals and the environment (4, 5), empirical domestic and foreign research on the physical and mental health of children with ASD as well as its health intervention has been carried out in an orderly manner. Although preliminary results have been achieved, the results are uneven, the scope of application is narrow and the promotion is poor (6–8). With the promulgation of national and social policies and regulations for special children and the increase in funding investment, physical intervention methods are also constantly developing. For example, a personalized teaching model based on physical activities [a teaching model that considers children’s motor abilities, personality traits, interests and hobbies (9)] has been launched in Peizhi schools in first-tier cities, such as Beijing and Shanghai. Even if this model adopts class teaching and one-to-one rehabilitation courses, how to accurately grasp the exercise needs of different types of children and how to build an efficient, fair and equal participation program still needs further exploration.

Accordingly, this two-participant case report explored descriptive within-case changes during a 16-week individualized exercise intervention for two children with ASD and co-occurring intellectual impairment. The study was intended to provide preliminary, exploratory, and hypothesis-generating information for the design of future controlled studies rather than to establish generalized intervention effectiveness.

## 2 Case presentation

Two children with autism spectrum disorder (ASD) were recruited from a special education school in Shanghai. Before enrollment, both had been diagnosed with ASD by qualified child psychiatrists at a medical institution according to DSM-5 criteria. Neither participant had received medication during the previous three months.

During the interview of guardians, Case B (pseudonym, male, 8.17 years old, delivered by caesarean section) was found by parents that the child was behind in language and social interaction at around 4 years old, and was diagnosed with ASD at 5 years old. No obvious abnormalities were found in his brain MRI scan, and the electroencephalogram (EEG) test showed that the indicators were within the normal range. As for the main manifestations, he unconsciously pronounced “Baba, mama” before 1 year old, and afterwards, his progress was slow, with occasional spontaneous speech or talking to himself. Although he could point to objects and selectively listen to instructions, he could not concentrate for a long time, sit still or stand still. He also knows simple numbers from 1 to 10, and has a good memory, and likes to flap hands and spit ptysis. Case D (pseudonym, female, 9.42 years old, born naturally) was diagnosed at 6 years old, and her genetic diagnosis of fragile X chromosome syndrome was normal. As for the main manifestations, most of her language skills are imitation, and occasionally she can write imaginative sentences. Moreover, she likes to flap head, shake hands and spit ptysis, but does not like to interact with his peers, nor share/show off.

For the observation of researchers, Case B can simply flip through books, but does not know why he flips through them, and also can fold simple triangles and paper airplanes. He gets nervous around strangers or crowded places, and avoids eye contact, while sometimes he walks around aimlessly. He has a good sense of music and can play the piano. Case D is used to sitting alone or going to the toilet frequently, for example, she goes to the toilet after every class, and if does not go, she will keep shouting or talking to herself. Occasionally, she can accept being forced not to go to the toilet. After going to the toilet, she likes to pull up his pants outside the toilet, and if any step is missing, she will go to the toilet again and repeat all the steps. She likes to pass and dribble the ball, but cannot dribble the ball at a fixed position, and the dribble height is low. Additionally, when participating in a favorite project, she would like actively to participate, but when encountering a more difficult project or there are many companions, she tends to escape and retreat. Being praised by familiar teachers is also one of her hobbies.

**Figure 1:**
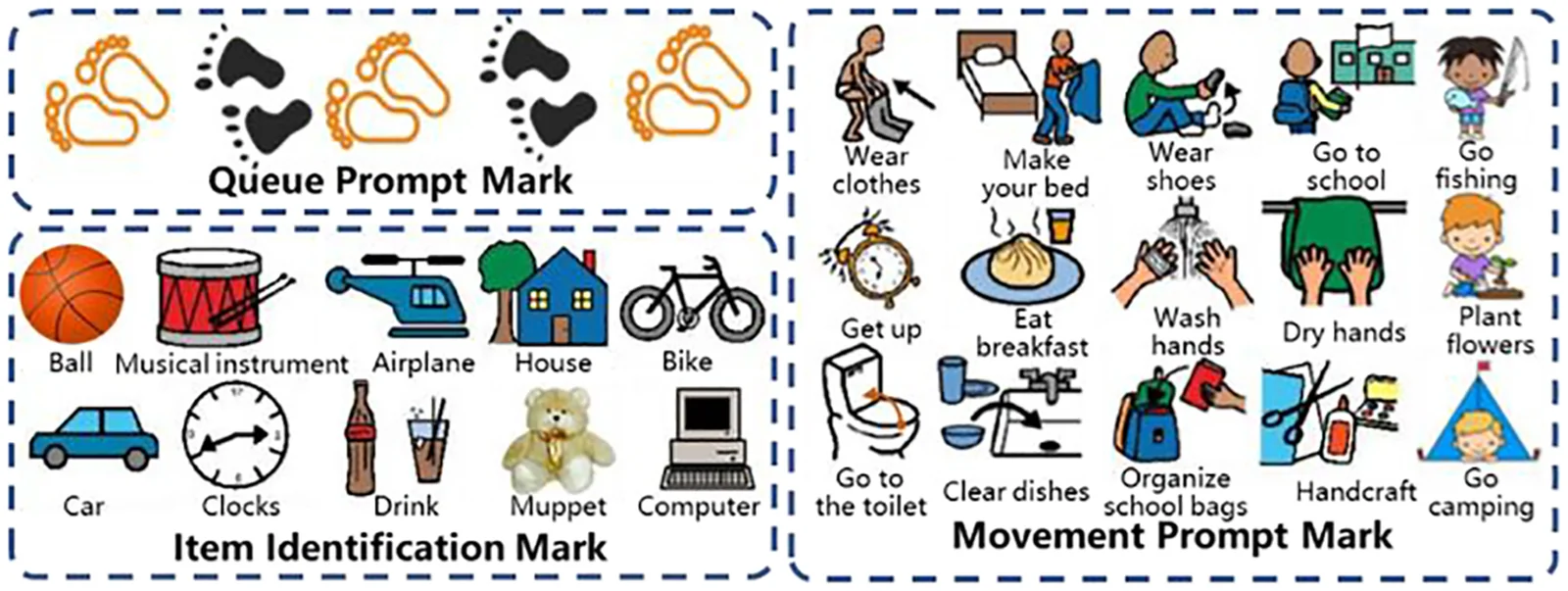
Prompt maks for children with ASD.

This study used a two-participant, cross-subject multiple-baseline case design. The 16-week duration and three-phase progression were developed with reference to medical training therapy principles, the 2016 Chinese curriculum standards for sports and health rehabilitation in special education schools, and previous exercise-intervention studies involving children with ASD. The program progressed from familiarization and locomotor activities (Weeks 1–5), to object-control skill practice and structured games (Weeks 6–10), and finally to skill consolidation and more independent task participation (Weeks 11–16). Visual prompt strategies, as shown in **Figure 1**, are used to attract children’s intentional attention, detailed activities are provided in **Supplementary Table 1**.This progression was intended to accommodate the participants’ learning characteristics and gradually reduce their dependence on prompts.

Sessions were delivered three times per week for 60 min by an adapted physical education teacher and a rehabilitation professional experienced in working with children with ASD. Exercise intensity and task difficulty were individualized according to physical capacity, behavioral tolerance, and task performance rather than a fixed physiological target. Progression was based on improved participation, task completion, reduced reliance on prompts, and more independent performance. Attendance and session completion were recorded, and intervention fidelity was monitored using standardized session plans, video recordings, and post-session review. No additional home-based exercise program was prescribed.

## 3 Results and discussion

During the basic training, intensive training and consolidation training stages, the physical activity (PA) of Case B and D decreased their sitting time as the intervention time prolonged, and their low-intensity physical activity (LPA), moderate- to vigorous-intensity physical activity (MVPA) and total physical activity (TPA) all increased as shown in **Table 1**.

**Table 1:** Changes in TGMD-3. HRPF and PA at different stages.

Cases	Items	Parameters	Week 1	Week 6	Week 11	Week 16
Case B	Physical activity	SB	533.27	443.62	398.36	243.2
		LPA	77.53	216.49	378.74	444.92
		MVPA	27.26	87.7	119.11	162.31
		TPA	104.79	304.19	497.85	607.23
	TGMD-3	Total score	3	5	8	7
		Locomotor skills	3	4	4	3
		Object control skills	0	1	4	4
	HRPF	BMI	14.19	14.55	13.13	13.49
		Waist-to-hip ratio	0.9	0.93	0.93	0.92
		Pacer 20m	2	4	6	6
		Vertical jump	5	5	6	9
		Grip strength	5.5	7.1	7.5	7.3
		1-minute sit-ups	3	6	5	5
		New sit-up	5.7	6	8	10
Case D	Physical activity	SB	432.74	421.87	394.25	214.58
		LPA	43.25	173.98	192.77	201.44
		MVPA	12.53	24.31	43.46	48.98
		TPA	55.78	98.29	236.23	250.42
	TGMD-3	Total score	4	6	10	14
		Locomotor skills	3	5	5	6
		Object control skills	1	1	5	8
	HRPF	BMI	18.23	15.15	14.27	13.31
		Waist-to-hip ratio	1.27	1.01	1.07	1.09
		Pacer 20m	0	1	1	3
		Vertical jump	3	4	3	4
		Grip strength	4.5	7.3	8.2	8.7
		1-minute sit-ups	0	1	2	3
		New sit-up	2.7	3.2	5.8	8.3

Across the four assessment points, both participants showed descriptive within-case changes in PA. For Case B, sedentary behavior (SB) decreased from 533.27 to 243.20 min/day (−290.07 min/day; −54.4%), while LPA, MVPA, and TPA increased by 367.39 min/day (+473.9%), 135.05 min/day (+495.4%), and 502.44 min/day (+479.5%), respectively. For Case D, SB decreased from 432.74 to 214.58 min/day (−218.16 min/day; −50.4%), while the corresponding activity measures increased by 158.19 min/day (+365.8%), 36.45 min/day (+290.9%), and 194.64 min/day (+348.9%). These values are descriptive and were not subjected to inferential significance testing.

In terms of HRPF, Case B and D showed improvement after exercise intervention. This is specifically reflected in the improvement in body composition (BMI), muscle strength and endurance (grip strength), and flexibility (forward bending in the new sitting position) as shown in **Table 1**. These outcomes may be relevant to body composition, muscular function, joint mobility, and participation in daily activities; however, no inferential statistical analysis or established minimal clinically important difference threshold was applied, and the changes should therefore be interpreted cautiously.

In terms of TGMD-3, Case B and D showed improvement after sports intervention, which was specifically reflected in the improvement in the total score and the sub-dimensions of object control skills, and there were also changes in displacement skills, as shown in **Table 1**. These descriptive changes may reflect improved movement competence and the ability to participate in school and recreational activities, but they do not establish a generalized intervention effect.

**Figure 2:**
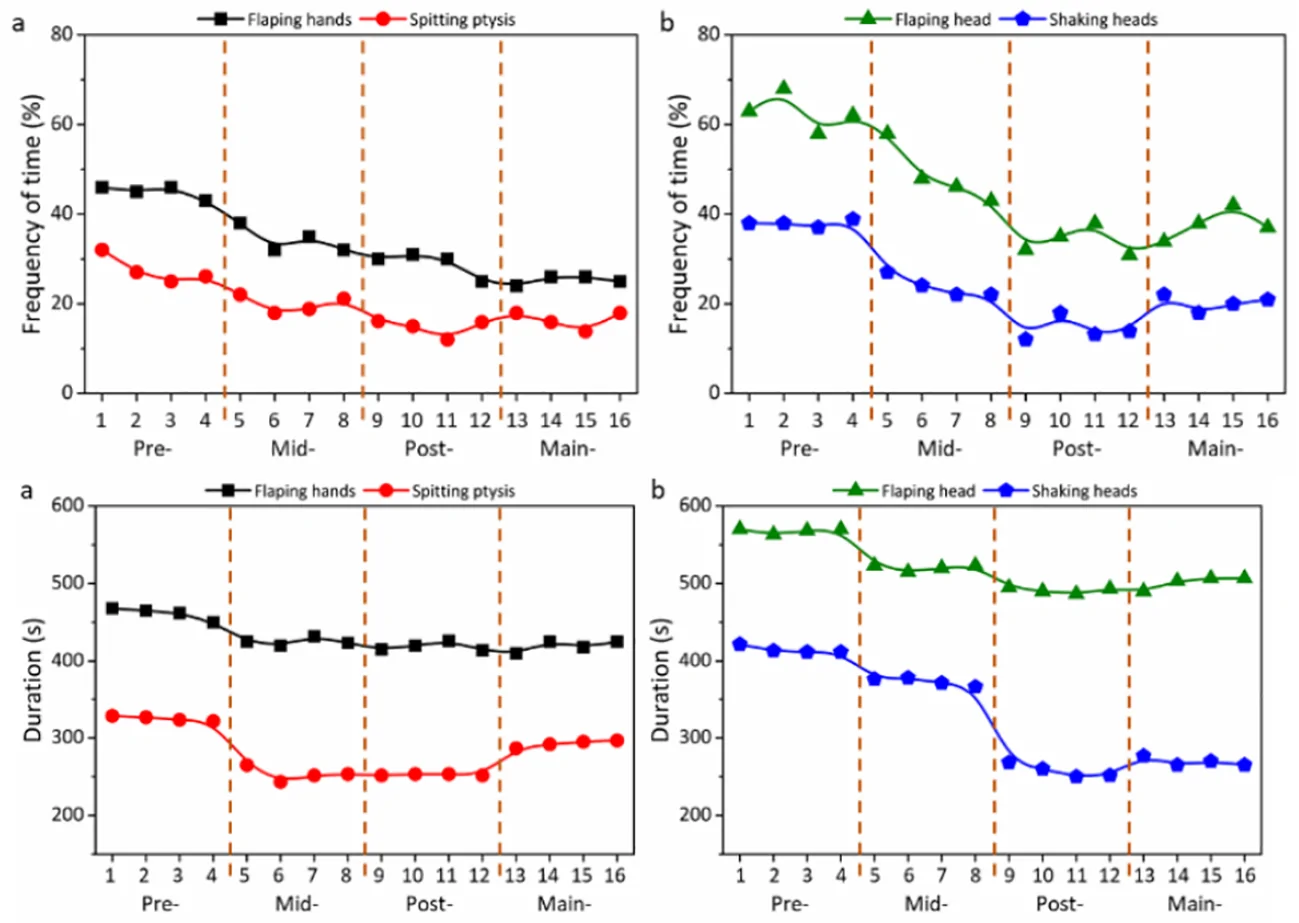
Frequency and duration of repetitive stereotyped behaviors for **(A)** Case B and **(B)** Case D during the intervention process.

**Figure 3:**
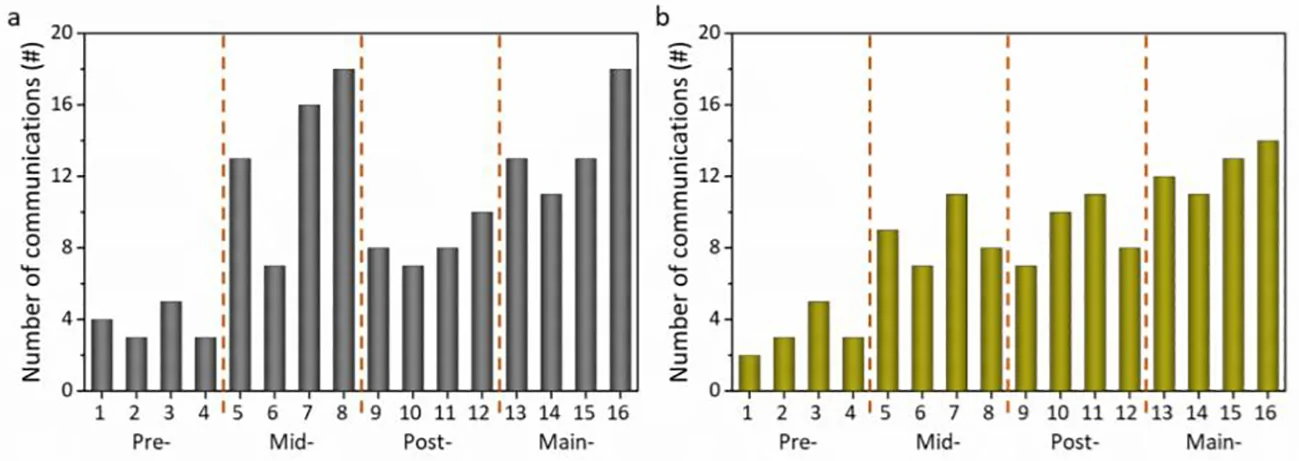
Positive communication times for **(A)** Case B and **(B)** Case D during the intervention process.

The observation method was used to analyze the changes in the frequency and duration of children’s behaviors at different stages. In terms of the repetitive stereotyped behaviors as shown in **Figure 2**, the frequencies of flapping hands and spitting ptysis for Case B were decreased by 18% and 17.7%, respectively, while the frequencies of flapping and shaking head for Case D were decreased by 25% and 17.7%, respectively. In terms of duration as shown in **Figure 3**, the durations of flapping hands and spitting ptysis for Case B were decreased by 24.7 s and 66.7 s, respectively, and the duration of flapping and shaking head for Case D were decreased by 32.3 s and 145.9 s, respectively. These patterns represent descriptive within-case trends and should not be interpreted as evidence of causality.

**Figure 3** shows the changes in the communication behaviors of Case B and D at different stages. It can be found that the average communication number of Case B is 9.81 times, and the number of positive communications increased from 3 times to 18 times, while the average communication number of Case D is 8.38 times, and the number of positive communications increased from 2 times to 14 times. Because communication behavior may also have been influenced by routine special education, teacher interaction, family support, and repeated observation, these changes are interpreted as exploratory rather than as evidence that the exercise intervention alone produced the outcome.

This two-case report examined descriptive within-case changes during a 16-week individualized exercise intervention. Both participants showed reduced sedentary time and increased physical activity, together with observable changes in selected fitness, motor, stereotyped-behavior, and communication outcomes. Given the design, these findings are interpreted as preliminary and hypothesis-generating.

The physical activity trends are consistent with previous case-based and group studies reporting that structured exercise can increase activity participation in children with ASD (10, 11). The repeated schedule, predictable session structure, individualized reinforcement, and gradual progression may have supported engagement in the present cases. Nevertheless, the magnitude of change differed between Cases B and D, which is consistent with the heterogeneity of ASD and suggests that participant characteristics and exercise dose may influence response.

The observed changes in grip strength, flexibility, and motor skills are also broadly consistent with previous adapted physical education and motor-skill reports (4, 5). Repeated practice and progressively more demanding locomotor and object-control tasks may have supported neuromuscular coordination and movement competence. Clinically, such changes could facilitate participation in daily, school, and recreational activities; however, because minimal clinically important difference thresholds were not available, clinical meaningfulness cannot be confirmed.

The reductions in selected stereotyped behaviors and increases in positive communication are compatible with recent meta-analytic and multidisciplinary evidence (12, 13), but the present findings are not directly comparable with controlled group studies. Possible explanations include behavioral substitution through structured movement, increased opportunities for communication, positive reinforcement, and enhanced teacher–child interaction (14). These mechanisms remain speculative because the study did not manipulate or independently measure them.

Routine special education and support from teachers and family members continued throughout the study and may have contributed to the behavioral and communication changes. Accordingly, the findings should not be attributed solely to exercise (15). The present observations mainly support the feasibility of individualized, progressively structured exercise and provide hypotheses for future controlled studies that standardize exercise dose, monitor concurrent services, and include longer follow-up.

## 4 Conclusions

During the 16-week individualized exercise intervention, Cases B and D showed descriptive reductions in sedentary behavior and increases in physical activity, together with observable changes in selected health-related fitness, fundamental movement skills, stereotyped behaviors, and positive communication. These observations are specific to the two participants and do not establish causal or generalized effectiveness. They provide preliminary, exploratory, and hypothesis-generating evidence that may inform the design of larger controlled studies of individualized exercise in educational rehabilitation settings.

## Data Availability

The original contributions presented in the study are included in the article/**Supplementary Material**. Further inquiries can be directed to the corresponding author.
