# Supplementary material for: Effects of a 16-week individualized exercise intervention on physical and behavioral outcomes in two children with autism spectrum disorder: a case series
Source: Front Psychiatry. 2026 Aug 17;17:1916839. doi: 10.3389/fpsyt.2026.1916839 (PMC13526954; doi:10.3389/fpsyt.2026.1916839)
Supplement: Supplementary file 1 [file SupplementaryFile1.doc]

**Supplementary Material**

**Effects of a 16-Week Individualized Exercise Intervention on Physical and Behavioral Outcomes in Two Children with Autism Spectrum Disorder: A Case Series**

**Yu Xing ^a,b,c^** (xingyu@hainanu.edu.cn)**, Haoyan Liu ^a^** (liuhaoyan@hainanu.edu.cn)**, Liang Li^d^, Xueping Wu ^c^，Qun Xiong ^e,*^** (441305881@qq.com)

^a^ *School of Physical Education, Hainan University, Haikou, Hainan 570228, China.*

^b^ *Hainan Provincial Key Laboratory of Sports and Health Promotion, Key Laboratory of Emergency and Trauma, Ministry of Education, The First Affiliated Hospital of Hainan Medical University, Hainan Medical University, Haikou 571199, China.*

^c^ *School of physical education and training, Shanghai University of Sport, Shanghai 200438, China*

^d^ *Jiangxi Normal University, Jiangxi, 330000, China*

^e^ *Jiangxi University of Chinese Medicine, Jiangxi, 330000, China; 441305881@qq.com (Q.X.)*

** Correspondence: xingyu@hainanu.edu.cn (Q.X)*

**1. Methods**

1.1. *Participants*

Written informed consent was obtained from their parents or legal guardians. Cognitive ability and autism symptom severity were assessed using the Wechsler Intelligence Scale for Children and the Childhood Autism Rating Scale (CARS) by trained professionals experienced in ASD and intellectual disability assessment. Case B had an intelligence score below 45 and a CARS score of 40, while Case D had an intelligence score below 45 and a CARS score of 38, indicating co-occurring intellectual impairment.

Physical activity, health-related fitness, and motor skills were assessed by trained researchers using standardized procedures, and behavioral outcomes were independently coded by trained evaluators. Changes in observed behaviors were treated as exploratory within-case outcomes rather than changes in diagnostic status.

According to guardian reports and school records, no new applied behavior analysis, speech and language therapy, occupational therapy, sensory integration therapy, pharmacological treatment, or other rehabilitation service was introduced during the 16-week intervention. Both participants continued their routine special education programs, which remained unchanged. These concurrent educational and family supports were considered potential contextual influences on the behavioral and communication outcomes.

1.2. Outcome Assessments

(1) Physical activity. The ActiGraph GT3X+ accelerometer was used to assess the daily sedentary, low-intensity, moderate-intensity, and high-intensity physical activity time of children. Before the test, the researchers initialized the accelerometer and set the test time. Then, they distributed the accelerometer to the children, demonstrated how to wear it, and explained the relevant precautions. The children were required to wear the accelerometer on their right hip for one week, during which their guardians were informed in the consent form that the children had to wear it from waking up in the morning to going to bed at night, except the bathing or swimming time. Afterwards, the researchers used ActiLife (Version 5.5.5) to export the raw data and screened the data based on the criteria that each data item included at least 5 school days and 2 weekend wearing days, and the wearing time was not less than 8 hours per day. Finally, Kinesoft software was used to extract, process, and analyze the physical activity data.

(2) Health-related physical fitness. Four domains were assessed: body composition (body mass index and waist-to-hip ratio), cardiorespiratory endurance (20-m Progressive Aerobic Cardiovascular Endurance Run), muscular strength and endurance (vertical jump, grip strength, and 1-min sit-ups), and flexibility (sit-and-reach).

(3) Fundamental movement skills. Regarding the assessment of fundamental movement skills, Chinese scholars^[1,2]^ employed the third version of the Test of Gross Motor Development 3 (TGMD-3) to test normal children of different age groups, and they found that the scale had good reliability and validity. Given that the TGMD-3 is applicable to children aged 3-10 years and its main test content includes displacement skills and object control skills^[3]^, the TGMD-3 was used to assess the motor abilities of the children. Physical activity, health and physical fitness were tested four times, including the 1^st^, 6^th^, 11^th^, and 16^th^ week.

1.3. Observation

(1) Scholars have used the “ABC Behavior Observation Record Form” in their research on repetitive stereotyped behaviors in autism, and the results show that its reliability and validity were good ^[4-7]^. Therefore, this study used the ABC Behavior Observation Scale as a tool to record the frequency and duration of repetitive stereotypes in individual children and evaluate their repetitive stereotyped behaviors. The behavioral observation was conducted 3 times/week, and the average value was used as the reported result.

(2) A self-compiled “Active Communication Behavior Observation Record Form” was used to record the method, frequency, content and behavioral function of the individual's active communication behavior during the baseline and intervention periods, respectively.

1.4. Interview

During the baseline period, teachers and guardians were interviewed to understand the basic situation, physical activities, health and fitness, motor development, preferences and interests of the children. During the intervention period, teachers were interviewed weekly to understand the classroom performance and teaching implementation of the children. After the intervention, a self-made semi-structured interview outline of “Social Perspective Interview” was used to investigate the satisfaction and acceptability of the intervention among teachers, teaching managers and parents.

1.5 Condition control

Because of the individualized and interactive nature of the exercise intervention, blinding of the participants and intervention instructors was not feasible. To reduce potential measurement bias, consistent lighting and temperature were maintained during all assessments, and standardized assessment procedures were followed. Appropriate instructional strategies, including task decomposition, verbal and visual prompts, music, and individualized reinforcers, were used to support participant engagement and adherence. Behavioral observations were conducted in natural educational settings and independently coded by trained evaluators. Inter-rater agreement reached 86%. Participant attendance, task completion, and classroom engagement were also recorded throughout the intervention. In addition, semi-structured interviews with parents, teachers, and relevant staff were audio-recorded to document the implementation process and the observed within-case changes.

**2. Tables**

Table S1. Exercise rehabilitation program

| Time/weeks | Contents |
| --- | --- |
| Preparation | Fixed-point standing and animal imitation warm-up exercises |
| Basic activities | Basic sports skills practice |
| 1st | (1) Direction differentiation: follow the rhythm of the music and distinguish up, down, left, right, front and back. (2) Neck movement: complete the exercises of looking up, lowering and turning the head with assistance. (3) Body rotation exercises: complete the exercises of standing still, turning left, right and back with assistance. (4) Physical fitness exercises: cart game. |
| 2nd | (1) Kicking exercises: complete the kicking action with assistance, and set obstacles or targets of different heights for practice. (2) Squatting exercises: complete fixed-point squatting and squatting exercises while marching under instructions. (3) Marching formation exercises: complete horizontal and vertical formation exercises together with the assistance of the teacher. (4) Physical fitness exercises: crawling games. |
| 3rd | (1) Familiar with running movements: coordinate upper and lower limbs, and experience the power of pushing the soles of the feet. (2) Basic running exercises. (3) Fun running exercises: set different obstacles, distances and directions, and children complete running activities such as running around obstacles and changing directions with the help of teachers. (4) Physical fitness exercises: complete running relay games together with the help of teachers. |
| 4th | (1) In-situ high jump: set obstacles of different heights, and children perform high jump exercises from standing and jumping with the help of teachers. (2) Single-leg jump: teachers set different fixed points, and children gradually complete single-leg jump fixed points with the help of teachers (including different directions, distances and heights). (3) Double-leg jump: coordinate both lower limbs, experience pushing the ground with both feet, and jump over obstacles of different heights and colors. (4) Physical fitness: frog jumping lotus leaf game (design lotus leaves of different sizes and distances, and children complete the jump with the help of teachers). |
| 5th | (1) Parallel steps: parallel steps left and right 1, 2, 3, 4 times. (2) Slide: imitate the crab’s horizontal slide 1, 2, 3, 4 times, and then do it in the opposite direction. (3) Forward slide: one foot in front of the other, swing the arms at the elbows, and focus on cultivating the awareness of the feet being in the air for a short time. (4) Crab crawl and pony slide games. |
| Ending part | (1) Relaxation: relax the body through meditation accompanied by music. (2) Stretching: mainly stretch the muscles involved in physical activities. (3) Fascia gun relaxation. The teacher reviews what has been learned through activities and rewards the children for their progress in the training process. |

Table S1. Exercise rehabilitation program

| Time/weeks | Contents |
| --- | --- |
| Preparation | Fixed-point standing, equipment placement, animal imitation warm-up exercises |
| Basic activities | Basic sports skills practice |
| 6th | (1) Ball-bouncing exercise: Bounce the ball in place, control the ball to bounce at the waist, and change the size and material of the ball (such as tennis, basketball and volleyball). (2) Bounce the ball while walking: Bounce the ball while walking, control the ball to bounce at the waist, and complete the passing action. (3) Bounce the ball in place & bounce the ball while walking: Complete 4 times of bouncing the ball in place, and then bounce the ball while walking, set different distances, different exercise modes (landing once, catching the rebound ball twice), and choose fixed-point and moving ball. (4) Physical fitness exercise: ground rolling, corner ball-bouncing game. |
| 7th | (1) Ball-kicking exercise: kick the ball in place with bare hands, kick the ball in place. (2) Kick the ball while walking: swing your arms while running and kick the ball, control the direction of the ball, and complete a fixed-point distance kick. (3) Kick the ball around obstacles: set obstacles in different positions, and kick the ball around obstacles while walking. (4) Physical fitness exercise: Complete the kicking relay game with the teacher quickly within the specified time. |
| 8th | (1) Ball throwing exercise: throw the ball at a fixed point and control the direction of the ball. (2) Throw the ball with obstacles: complete the throwing action when passing through obstacles. (3) Continuous fixed point + moving throwing: throw the ball by setting different obstacles. (4) Physical fitness exercise: record the number of balls thrown within the specified time. |
| 9th | (1) Barehand one-handed hitting: hold the racket with one hand, adjust the racket face, imagine hitting the ball, and practice the complete action of swinging. (2) Fixed-point one-handed hitting: experience the sense of success of hitting the ball with the help of the teacher through the fixed-point height, and encourage children to complete the hitting action independently through retreat assistance. (3) One-handed hitting: hold the ball with one hand and the racket with the other hand, and perform the continuous action of throwing and hitting the ball with the help of the teacher, and control the direction of hitting the ball to face forward. (4) Physical fitness exercise: batting relay game. |
| 10th | (1) Barehand two-handed hitting: hold the racket with both hands, adjust the racket face, imagine hitting the ball, and practice the complete action of swinging. (2) Fixed-point two-handed hitting: Through the fixed-point height, with the assistance of the teacher, experience the sense of success in hitting the ball, and encourage children to complete the hitting action independently through retreat assistance. (3) Two-handed hitting: Through the fixed-point height toss the ball, with the assistance of the teacher, perform the continuous action of throwing and hitting the ball, control the direction of hitting the ball to face forward, and at the same time, you can choose different directions to practice two-handed hitting. (4) Physical fitness exercise: fixed-point hitting game. |
| Ending part | (1) Relaxation: Accompanied by music, relax the body through meditation. (2) Stretching: Mainly stretch the muscles involved in physical activities. (3) Fascia gun relaxation. The teacher reviews and rewards what has been learned through activities. |

Table S1 Exercise rehabilitation program

| Time/weeks | Contents |
| --- | --- |
| Preparation | Fixed-point standing, equipment placement, animal imitation warm-up exercises |
| Basic activities | Basic sports skills practice |
| 11th | (1) Running games: repeat the running exercises in Stage 2. (2) Alternate walking and running. (3) Walk, run and cross obstacles. (4) Physical fitness exercises: rapid arm swing and leg lifting exercises. |
| 12th | (1) Jumping games: repeat the jumping exercises in Stage 2. (2) Alternate walking, running and jumping. (3) Continuous walking, running and jumping exercises. (4) Physical fitness exercises: continuous high jumps and rapid running and jumping mixed exercises. |
| 13th | (1) Sliding games: repeat the sliding exercises in Stage 2. (2) Alternate walking, running, jumping and sliding. (3) Continuous walking, running, jumping and sliding exercises. (4) Physical fitness exercises: complete the corresponding exercises within the specified time. |
| 14th | (1) Ball-bouncing games: repeat the ball-bouncing exercises in Stage 2. (2) Bounce the ball while walking (walking or running). (3) Complete the ball bouncing at the specified number of times and height. (4) Physical fitness exercises: continuous ball hitting, ball hitting over obstacles, changing the route of ball hitting, etc. |
| 15th | (1) Throwing games: repeat the throwing exercises in Stage 2. (2) Throwing exercises while walking (running). (3) Throwing the ball alternately with the upper hand and the lower hand. (4) Physical fitness exercises: continuous (walking, running) throwing mixed exercises. |
| 16th | (1) Batting games: repeat the single-handed and double-handed batting games in Stage 2. (2) Practice the number and target of single-handed/double-handed batting. (3) Mixed single-handed and double-handed batting exercises. (4) Physical fitness exercises: continuous (single-handed and double-handed) batting mixed exercises. |
| Ending part | (1) Relaxation: relax the body through meditation accompanied by music. (2) Stretching: mainly stretch the muscles involved in physical activities. (3) Fascia gun relaxation. The teacher reviews and rewards what has been learned through activities. |

Table S2 Changes in TGMD-3. HRPF and PA at different stages

|  |  | Week 1 | Week 6 | Week 11 | Week 16 |
| --- | --- | --- | --- | --- | --- |
| Case B | Physical Activity  SB  LPA  MVPA  TPA  TGMD-3  Total score  Locomotor skills  Object control skills  HRPF  BMI  Waist-to-hip ratio  Pacer 20m  Vertical jump  Grip strength  1-minute sit-ups  New sit-up | 533.27  77.53  27.26  104.79  3  3  0  14.19  0.9  2  5  5.5  3  5.7 | 443.62  216.49  87.7  304.19  5  4  1  14.55  0.93  4  5  7.1  6  6 | 398.36 378.74  119.11 497.85  8  4  4  13.13  0.93  6  6  7.5  5  8 | 243.2  444.92  162.31  607.23  7  3  4  13.49  0.92  6  9  7.3  5  10 |
| Case D | Physical Activity  SB  LPA  MVPA  TPA  TGMD-3  Total score  Locomotor skills  Object control skills  HRPF  BMI  Waist-to-hip ratio  Pacer 20m  Vertical jump  Grip strength  1-minute sit-ups  New sit-up | 432.74  43.25  12.53  55.78  4  3  1  18.23  1.27  0  3  4.5  0  2.7 | 421.87  173.98  24.31  98.29  6  5  1  15.15  1.01  1  4  7.3  1  3.2 | 394.25  192.77  43.46  236.23  10  5  5  14.27  1.07  1  3  8.2  2  5.8 | 214.58  201.44  48.98  250.42  14  6  8  13.31  1.09  3  4  8.7  3  8.3 |

**3. Limitations**

This study has several important limitations. The sample comprised only two purposively selected participants, and there was no control group; therefore, selection bias, individual variability, and limited external validity restrict generalizability. Participants and intervention providers could not be blinded, and behavioral outcomes may have been affected by observer expectancy despite independent coding and inter-rater checks. Routine special education and family support continued during the intervention and may have confounded behavioral and communication outcomes. In addition, the study lacked long-term follow-up and did not use inferential statistics or established minimal clinically important difference thresholds. Consequently, causal relationships cannot be established. Future studies should use larger samples, controlled designs, blinded outcome assessment where feasible, standardized monitoring of concurrent services, and longer follow-up.

**Reference**

[1] Dong LS, Fan R, Shen B, et al. A comparative study on fundamental movement skills among children with autism spectrum disorder and typically developing children aged 7-10. Front Psychol.(2024),15: 1287752.doi:10.3389/fpsyg.2024.1287752

[2] Diao Y, Dong C, Li J. The establishment of norm of gross motor development test in shanghai. China Sport Science and Technology. (2018), 054(002): 99-105.

[3] Zhou Z, Peng D, Peng P, et al. Exercise Dose Architecture as a Precision-Informed Developmental Framework in Autism Spectrum Disorder: A Narrative Review of Mechanisms, Sex-and Development-Related Heterogeneity, and Clinical Translation. Front Psychiatry, (2026),17: 1865515.

[4] Xiao J. The Intervention study on autistic stereotyped behavior with positive behavior support. ChongQing Normal University, 2015.

[5] Tian X. A case study of fun running on stereotypic behavior of autism children. Guangzhou Sport University, 2017.

[6] Zan F, Xie A. A case study offunctional behavioral assessment of an autistic child. Chinese Journal of Special Education, (2007), (05): 62-67.

[7]Jozkowski AC, Lichtenwalner MA, Cermak SA. Case studies on the feasibility of exergaming to enhance physical activity in youths on the autism spectrum. GAP, (2016), 17(2): 24-36.
